# SPRY4 is responsible for pathogenesis of adolescent idiopathic scoliosis by contributing to osteogenic differentiation and melatonin response of bone marrow-derived mesenchymal stem cells

**DOI:** 10.1038/s41419-019-1949-7

**Published:** 2019-10-23

**Authors:** Jing Li, Na Li, Yunfei Chen, Shangyi Hui, Junfen Fan, Buqing Ye, Zusen Fan, Jianguo Zhang, Robert Chunhua Zhao, Qianyu Zhuang

**Affiliations:** 1 Center of Excellence in Tissue Engineering, Institute of Basic Medical Sciences and School of Basic Medicine, Chinese Academy of Medical Sciences and Peking Union Medical College, Beijing Key Laboratory of New Drug Development and Clinical Trial of Stem Cell Therapy, Beijing, P.R. China; 2 Department of Anesthesiology, Peking Union Medical College Hospital, Beijing, P.R. China; 3 CAS Key Laboratory of Infection and Immunity, CAS Center for Excellence in Biomacromolecules, Institute of Biophysics, Chinese Academy of Sciences, Beijing, China; 4 Department of Orthopedics, Peking Union Medical College Hospital, Beijing, P.R. China

**Keywords:** Kinases, Mesenchymal stem cells

## Abstract

Adolescent idiopathic scoliosis (AIS) is a complex, three-dimensional deformity of the spine that commonly occurs in pubescent girls. Decreased osteogenic differentiation and aberrant melatonin signalling have been demonstrated in mesenchymal stem cells (MSCs) from AIS patients and are implicated in the pathogenesis of AIS. However, the molecular mechanisms underlying these abnormal cellular features remain largely unknown. Our previous work comparing gene expression profiles between MSCs from AIS patients and healthy controls identified 1027 differentially expressed genes. In the present study, we focused on one of the most downregulated genes, SPRY4, in the MAPK signalling pathway and examined its role in osteogenic differentiation. We found that SPRY4 is markedly downregulated in AIS MSCs. Knockdown of SPRY4 impaired differentiation of healthy MSCs to osteoblasts, while SPRY4 overexpression in AIS MSCs enhanced osteogenic differentiation. Furthermore, melatonin treatment boosted osteogenic differentiation, whereas SPRY4 ablation ablated the promotional effects of melatonin. Moreover, SPRY4 was upregulated by melatonin exposure and contributed to osteogenic differentiation and melatonin response in a MEK-ERK1/2 dependent manner. Thus, loss of SPRY4 in bone marrow derived-MSCs results in reduced osteogenic differentiation, and these defects are further aggravated under the influence of melatonin. Our findings provide new insights for understanding the role of melatonin in AIS aetiology and highlight the importance of MSCs in AIS pathogenesis.

## Introduction

Adolescent idiopathic scoliosis (AIS) is a complex, three-dimensional deformity of the spine that commonly occurs during the peripubertal period aged 10–16 years^1,2^. The cause and pathogenesis of AIS remain obscure, but increasing reports demonstrate that AIS patients have abnormal skeletal growth^3^ and persistent lower bone mineral density (BMD)^4,5^. Additionally, previous studies have suggested that relative anterior overgrowth with disproportionate endochondral‑membranous bone growth contributes to the development of AIS^6^. Among the various theories for AIS aetiology involving biomechanical, neuromuscular, genetic and environmental origins^7^, melatonin is attracting increasing attention.

Melatonin, an indolamine produced primarily by the pineal gland, has been proven to regulate osteogenesis and osteolysis^8^. It can also exert bone-protective effects by reducing age-related bone loss and improving BMD^5,9^. Numerous studies reported that melatonin deficiency induces scoliosis in pinealectomized chickens^10,11^, bipedal rats^12^ and C57BL/6J mice^13^. Although it remains controversial whether there are differences in serum melatonin levels in human AIS patients^14,15^, melatonin signalling was found to be impaired in AIS patients^16,17^. Melatonin has also been reported to play an important role in the regulation of osteogenic and chondrogenic differentiation of human mesenchymal stem cells (MSCs)^18,19^. Furthermore, AIS MSCs exhibit abnormal cellular responses to melatonin^20^.

MSCs, which are multipotent precursors, can differentiate into chondrocytes, adipocytes or osteoblasts^21,22^. Endochondral and intramembranous ossification begin with MSC condensation and developmental programmes towards chondrogenesis and osteoblastogenesis^23,24^. MSCs from AIS patients have reduced osteogenic differentiation abilities compared to age- and gender-matched controls^25^. Differential proteome^26^ and gene expression profiles^27^ in AIS-MSCs shown by our team further revealed that dysregulated proliferation and developmental signalling pathways exist in AIS-MSCs. Recently, we identified a novel long noncoding RNA, lncAIS, that is downregulated in AIS MSCs and implicated in AIS pathogenesis^28^.

Given these findings, impaired melatonin signalling pathways in AIS patients and abnormal cellular responses of MSCs to melatonin, together with increasing evidence of AIS-MSCs anomalies, strongly suggest that MSC abnormalities play a critical role in AIS pathogenesis and accompanying osteopenia. The present study was conducted to provide further understanding and evidence of the molecular mechanism of reduced osteogenic differentiation and abnormal melatonin responses in AIS-MSCs. Herein, we show that SPRY4, a regulator of the receptor tyrosine kinase (RTK) signalling cascade^29^, promotes osteogenic differentiation of MSCs and is involved in melatonin-mediated pathogenesis in AIS.

## Material and methods

### Antibodies and reagents

Antibodies against ALP (ab108337) and OPN (ab69498) were purchased from Abcam (Cambridge, MA, USA). Antibodies against IBSP (5468), ERK1/2 (4370), p-ERK1/2 (4695), MEK1/2 (8727) and p-MEK1/2 (9154) were purchased from Cell Signaling Technology (Danvers, USA). The RUNX2 antibody (sc-390715) was purchased from Santa Cruz Biotechnology (Santa Cruz, CA, USA). Antibodies against SPRY4 (22765-1-AP), GAPDH (10494-1-AP) and β-actin (HRP-60008) were purchased from Proteintech (Wuhan, China).

Melatonin was purchased from Sigma-Aldrich (St. Louis, MO, USA). U0126 was purchased from Selleck Chemicals (Houston, TX, USA).

### Patients and specimens

Bone marrow (BM) aspirates were obtained from 23 AIS patients and 12 healthy controls. In the AIS group, 23 patients with a mean age of 14.3-years-old (ranging from 13- to 17-years-old) underwent full clinical and radiological examinations to rule out other causes of scoliosis and to ascertain the diagnosis of AIS. In the control group, twelve age- and sex-matched subjects, with a mean age of 14.6-years-old (ranging from 13- to 17-years-old) all had a straight spine and a normal forward bending test on physical examination. Participants in the control group were all confirmed to be free of any associated medical diseases or spinal deformities when entered into the study. This study was approved by the Ethics Committee of Peking Union Medical College Hospital. Written informed consent was obtained from all subjects and their parents before enrolling participants into the study.

### Isolation and culture of BM-MSCs

BM-MSCs were isolated and expanded as previously described^27^. Briefly, bone marrow tissues were collected from donors who underwent liposuction surgery and were cultured in DMEM/F-12 growth medium supplemented with 2% foetal bovine serum (FBS, Gibco), 1× Insulin-Transferrin-Selenium (ITS, Gibco), 10 ng/mL EGF (Peprotech), 10 ng/mL PDGF (Peprotech), 50 μM β-mercaptoethanol (Sigma), 2 mM l-glutamine (Invitrogen) and 100 U/mL penicillin and 100 μg/mL streptomycin.

### Osteogenic differentiation of MSCs

To induce osteogenic differentiation, MSCs from passage 2–5 were seeded into six-well plates to 60–80% confluence and then supplemented with osteogenesis induction medium containing high-glucose DMEM supplemented with 10% FBS, and 10 mM β-glycerophosphate (Sigma) and 0.2 mM ascorbic acid (Sigma). Medium was changed every other day during osteogenic differentiation.

For comparison of osteogenic differentiation between SPRY4 knockdown/overexpression and corresponding controls, MSCs from at least three donors were used, and all experiments were replicated at least three times. To test the effect of melatonin on osteogenic differentiation, 100 μM melatonin (Sigma) or DMSO was added to the osteogenesis induction medium. To verify the effects of the MAPK pathway on osteogenic differentiation, different concentrations (10 or 50 μM) of U0126 (Selleck) were added to osteogenesis induction medium.

### RNA extraction and quantitative reverse transcription-polymerase chain reaction (qRT-PCR)

Total RNA was extracted using TRIzol reagent (Invitrogen) and was treated with DNase I (Ambion) at 37 °C for 30 min^30^. Reverse transcription was performed using a Reverse Transcription kit (Takara, Japan), and qRT-PCR was performed with HieffTM qPCR SYBR® Green Master Mix (Yeasen, China) using a Step One Plus Detection System (Applied Biosystems, USA). Relative expression levels of mRNA were evaluated using the 2^−ΔΔCt^ method and were normalized to the expression of *GAPDH*. Primers sequences are shown in Supplementary Table 1.

### Western blot analysis

Cells were lysed in RIPA lysis buffer (Beyotime) with 1 mM PMSF on ice for 30 min and were manually scraped from culture plates. Protein samples were quantified using the BCA assay Kit (Beyotime), separated using 10% SDS-PAGE, and then transferred onto polyvinylidene difluoride (PVDF) membranes (0.45 μm, Millipore). The membranes were blocked with 5% BSA for 1 h at room temperature and incubated with primary antibodies overnight at 4 °C. After washing several times, membranes were incubated with horseradish peroxidase (HRP)-conjugated secondary antibodies (Zhongshan, China) for 1 h at room temperature. Proteins were detected using a chemiluminescent ECL reagent (Millipore).

### Alkaline phosphatase staining and relative alkaline phosphatase activity assays

Alkaline phosphatase (ALP) staining was performed according to the manufacturer’s instructions (Institute of Haematology and Blood Diseases Hospital, Chinese Academy of Medical Sciences, Tianjin, China). For relative ALP activity assays, cells were washed twice with PBS and lysed in RIPA lysis buffer (Beyotime) with 1 mM PMSF. After centrifugation and quantification, 5 μl protein lysate were incubated with 200 μl Alkaline Phosphatase Yellow Liquid Substrate System (pNPP, Sigma) in 96-well plates for 30 min at 37 °C. ALP activity was measured photometrically at 405 nm using a spectrophotometer and normalized to the protein concentration of cell lysates^30^.

### Alizarin red S staining and quantification

Alizarin red staining was performed to detect calcium salt deposition in the later stages of bone formation. Briefly, cells were washed twice with PBS and fixed with 95% ethanol for 10 min. Then, cells were washed with double-distilled water and stained using 0.5% Alizarin red solution with a pH of 4.2 for 30 min at room temperature. Cells were then washed with double-distilled water to remove unbound dye and were imaged by light microscopy. For quantification of Alizarin red S staining, 300–500 μL of 10% (w/v) cetylpyridinium chloride (Sigma) were added to each well. The plate was shaken until the Alizarin red was completely dissolved. The resultant solution was transferred to the 96-well plate, and absorbance was measure at a wavelength of 570 nm.

### siRNAs transfection and virus infection

siRNAs used to knockdown SPRY4 mRNA and negative control were designed by the online tool (BLOCK-iT™ RNAi Designer) and synthesized by GenePharma company (China) (listed in Supplementary Table 1). For transfection of siRNAs or plasmids, Lipofectamine 2000 (Life Technology) was used according to the manufacturer’s recommendations. For stable knockdown, lentivirus shRNA expression vector targeting the same sequences as siRNAs were constructed and packaged by GenePharma Company (China). For overexpression, the coding sequence (CDS) of SPRY4 was inserted into a lentivirus vector (pEZ-Lv225) and packaged by GeneCopoeia^TM^ Company (China). MSCs with stable SPRY4 knockdown, overexpression and corresponding controls were generated by lentivirus infection (MOI = 10) for 24 h, followed by selection using 1 μg/ml puromycin.

### Heterotopic bone formation in vivo

MSCs with SPRY4 knockdown, SPRY4 overexpression or corresponding controls (stably transduced with lentivirus vectors) were incubated in osteogenic medium for 3 days. Then, 2 × 10^6^ cells were loaded in Bongold^®^ (purchased from Beijing Allgens Medical Science and Technology Co., Ltd.), an artificial biomimetic mineralized collagen scaffold, incubated at 37 °C overnight and then implanted subcutaneously into the upper dorsal surface of 8-week-old NOD/SCID mice (*n* = 6 for each group). After 10 weeks, implants were harvested and fixed in 4% paraformaldehyde, decalcified in 10% EDTA and embedded in paraffin. Osteoid formation was observed using HE staining, and collagen synthesis was detected by Masson staining. All staining procedures were performed according to the manufacturer’s protocol from ServiceBio (http://www.servicebio.cn/)^30^. Scaffolds without cell loading were implanted as negative controls, and tibia from mice were use as positive controls for histological analysis. All animal experiments were performed in accordance with guidelines and permissions of the Ethics Committee of the Chinese Academy of Medical Sciences and Peking Union Medical College.

### Statistical analysis

Data are presented as the means ± S.D. Significant differences between groups were analysed by unpaired Studentʹs *t*-tests. Differences were considered statistically significant at *p < 0.05, **p < 0.01 and ***p < 0.001.

## Results

### *SPRY4* is downregulated in AIS MSCs and is induced by melatonin exposure

**Fig. 1:**
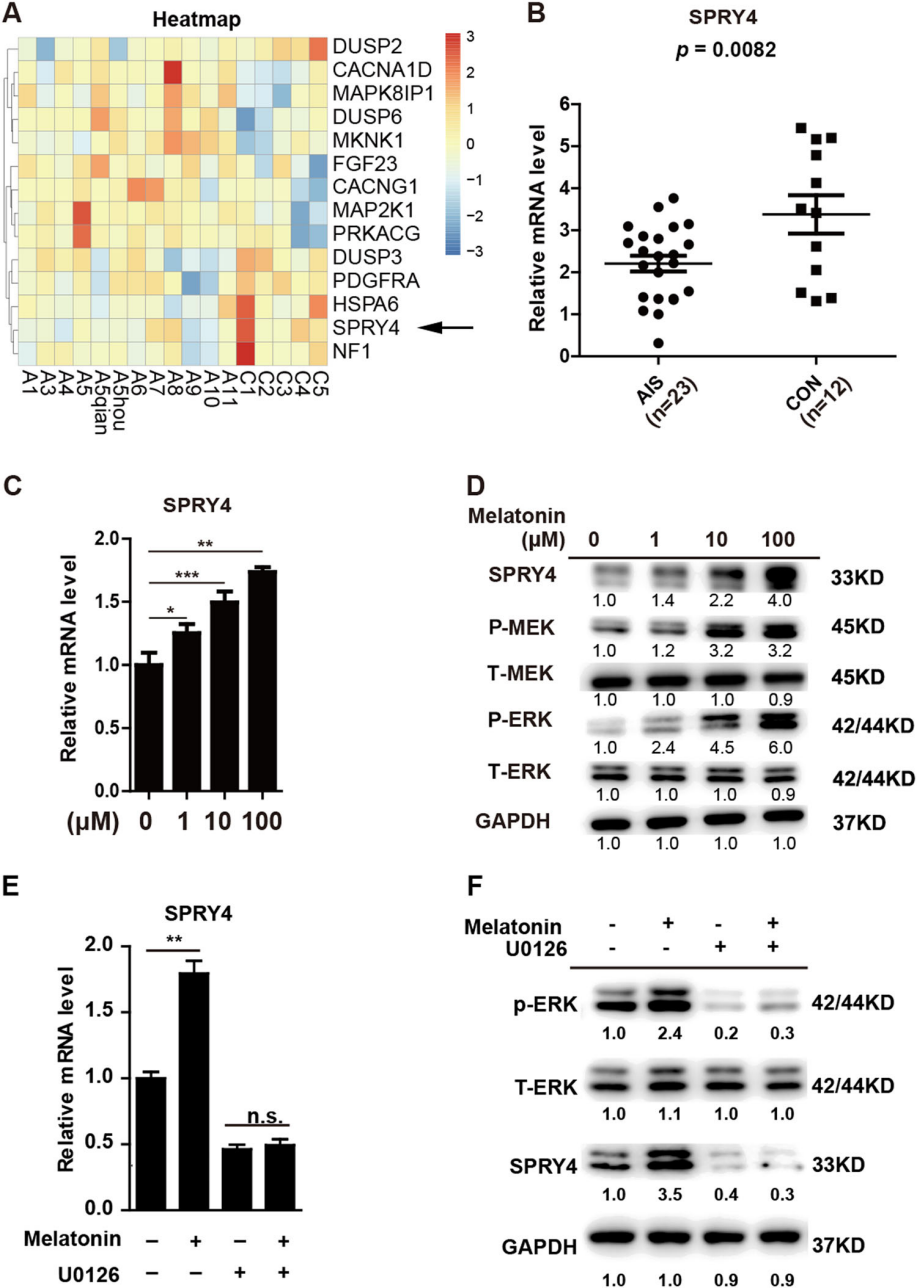
SPRY4 exhibits reduced expression in AIS BM-MSCs and is regulated by melatonin. **a** Hierarchical clustering of genes in MAPK signalling pathway that were differentially expressed in BM-MSCs between AIS patients (*n* = 10) and healthy individuals (*n* = 5). Black arrowhead denotes SPRY4. **b** Expression of *SPRY4* in BM-MSCs from AIS patients (*n* = 23) and healthy individuals (*n* = 12) was analysed by qRT-PCR. Primer sequences are listed in Supplementary Table 1. **c** MSCs were treated with various concentrations (0, 1, 10, 100 μM) of melatonin for 48 h. Then, expression of *SPRY4* was determined by qRT-PCR. Data were from three independent experiments using BM-MSCs derived from three healthy donors. **d** Representative western blot of SPRY4, total-MEK1/2 (T-MEK), phospho-MEK1/2 (P-MEK), total-ERK1/2 (T-ERK) and phospho-ERK1/2 (P-ERK) was detected. GAPDH was used as a loading control in both qRT-PCR and western blot analysis. Data are shown as the means ± SD. **e** BM-MSCs were treated with melatonin (100 μM) and U0126 (10 μM) for 48 h. Expression of SPRY4 was detected by qRT-PCR. **f** P-ERK, T-ERK, SPRY4 and GAPDH were analysed by western blot. Data were from three independent experiments using BM-MSCs derived from three healthy donors

In our previous study, we compared gene expression patterns of BM-MSCs from five healthy donors and 10 AIS patients using microarray analysis and found 1027 differentially expressed genes (DEGs)^27^. Pathway analysis revealed that mitogen-activated protein kinase (MAPK) signalling, which plays a crucial role in both osteogenic differentiation^31,32^ and melatonin response^33–35^, is significantly dysregulated in AIS MSCs^27^. Thus, we reasoned that DEGs in the MAPK pathway might contribute to aberrant osteogenic differentiation and melatonin responses in AIS MSCs. Among 14 DEGs in the MAPK pathway (Fig. 1a), SPRY4, a negative regulator of the bFGF/MAPK pathway, displayed the most significant downregulation (fold change = 0.79, *p* = 0.001866).

To examine the potential correlation between SPRY4 and AIS, we performed qRT-PCR in BM-MSCs collected from AIS patients and healthy donors (*n* = 23 and 12, respectively). In line with previous microarray data, AIS MSCs exhibited decreased *SPRY4* expression compared to healthy MSCs (*P* = 0.0082) (Fig. 1b). Next, we treated MSCs with increasing concentrations of melatonin (0, 1, 10 and 100 μM) and examined expression of *SPRY4* by qRT-PCR. As shown in Fig. 1c, *SPRY4* is upregulated by melatonin in a concentration-dependent manner. Western blot also demonstrated that the SPRY4 protein is dramatically elevated in response to melatonin (Fig. 1d). Consistent with previous reports, melatonin exposure increased phosphorylation levels of MEK1/2 and ERK 1/2 in MSCs. We incubated BM-MSCs with the ERK inhibitor U0126 in the presence of melatonin. We found that U0126 could abolish the upregulation of SPRY4 induced by melatonin (Fig. 1e, f). These data suggest that SPRY4 is downregulated in AIS MSCs and might be involved in melatonin signalling.

### Knockdown of SPRY4 impairs osteogenic differentiation of MSCs

**Fig. 2:**
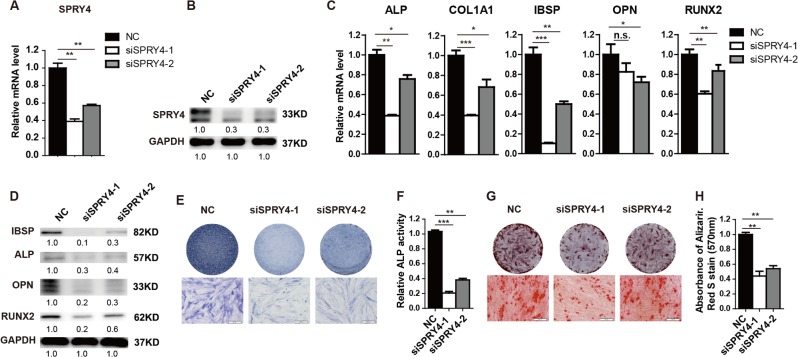
Knockdown of SPRY4 impairs osteogenic differentiation. **a**, **b** SPRY4 was silenced in BM-MSCs from healthy donors by two independent siRNAs (siSPRY4-1 and siSPRY4-2). Knockdown efficiency was verified by qRT-PCR (**a**) and western blot (**b**). **c** qRT-PCR analysis detected osteogenic transcription factors and marker genes *ALP*, *COL1A1*, *IBSP*, *OPN* and *RUNX2* on day 6 of osteogenic differentiation. Data were from three independent experiments using BM-MSCs derived from three healthy donors. **d** Western blot analysis detected osteogenic transcription factors and marker genes IBSP, ALP, OPN and RUNX2 on day 6 of osteogenic differentiation. Data were from three independent experiments using BM-MSCs derived from three healthy donors. **e**, **f** ALP staining and relative ALP activity assays were performed on day 6 of osteogenic differentiation. Data were from three independent experiments using BM-MSCs derived from three healthy donors. **g**, **h** Calcium deposition by Alizarin red S staining and quantification were performed on day 12 of osteogenic differentiation. Data were from three independent experiments using BM-MSCs derived from three healthy donors. GAPDH was used as a loading control in both qRT-PCR and western blot analyses. Data are shown as the means ± SD

To investigate the role of SPRY4 in MSC differentiation, we silenced expression of *SPRY4* in healthy BM-MSCs using two independent siRNAs. Knockdown efficiency was confirmed by qRT-PCR (Fig. 2a) and western blot (Fig. 2b) compared to the negative control (NC). Then, we induced MSCs to differentiate into the osteogenic lineage and measured expression of osteogenic transcription factors and marker genes at both mRNA (Fig. 2c) and protein (Fig. 2d) levels. During osteogenic differentiation, expression of *ALP*, *COL1A*1, *IBSP, OPN* and *RUNX2* were significantly decreased in SPRY4-depleted cells compared to controls. Repressed ALP staining and ALP activity (Fig. 2e, f), as well as reduced mineral deposition detected by Alizarin red staining (Fig. 2g, h), further indicated that SPRY4 knockdown impairs osteogenic differentiation of MSCs.

### Ectopic expression of SPRY4 improves osteogenic differentiation of AIS MSCs

**Fig. 3:**
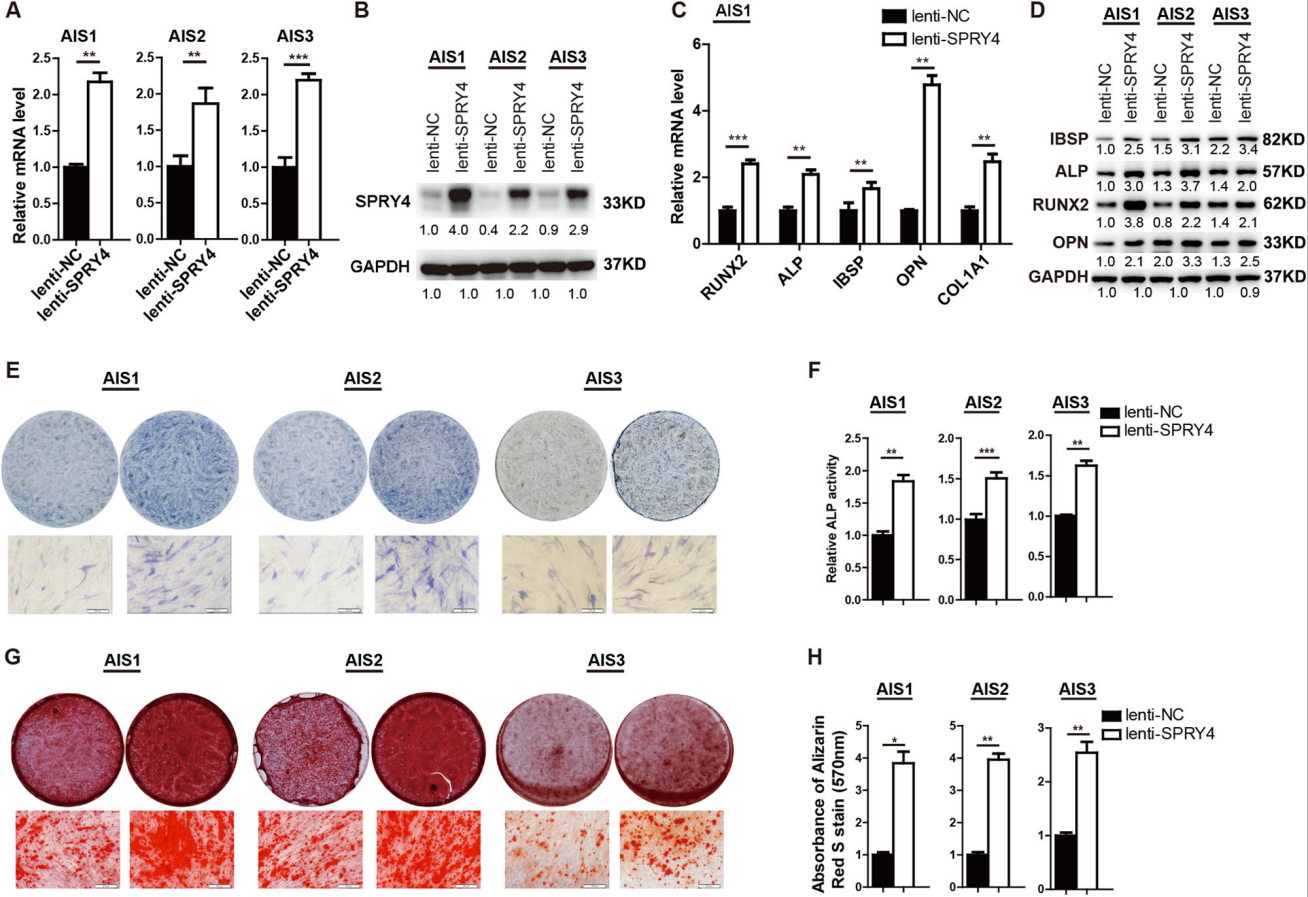
Ectopic expression of SPRY4 improves osteogenic differentiation of AIS MSCs in vitro. **a**, **b** AIS BM-MSCs from three patients were transduced with lentivirus overexpressing SPRY4 (lenti-SPRY4) or empty vector (lenti-NC). Efficiency of ectopic expression was verified by qRT-PCR (**a**) and western blot (**b**). **c** qRT-PCR analysis of osteogenic transcription factors and marker genes *RUNX2*, *ALP*, *IBSP*, *OPN* and *COL1A1* on day 6 of osteogenic differentiation. **d** Western blot detected osteogenic transcription factors and marker genes IBSP, ALP, RUNX2 and OPN on day 6 of osteogenic differentiation. **e**, **f** ALP staining and relative ALP activity assays were performed on day 6 of osteogenic differentiation. **g**, **h** Calcium deposition by Alizarin red S staining and quantification were performed on day 12 of osteogenic differentiation. Data were from three independent experiments using BM-MSCs derived from three AIS patients. GAPDH was used as a loading control in both qRT-PCR and western blot analyses. Data are shown as the means ± SD

Previous studies have indicated that osteogenic differentiation abilities and alkaline phosphatase activities of AIS MSCs are lower compared to age- and gender-matched control^25^. In this study, we first verified these results by comparing osteogenic differentiation of MSCs from three AIS patients and three healthy donors. As shown in Supplementary Fig. 1, ALP expression and activity, as well as mineral deposition, were relatively low in AIS MSCs after osteogenic induction. Then, we transduced the AIS MSCs mentioned above with lentivirus to determine whether overexpression of SPRY4 could restore the defects observed in osteogenic differentiation. In AIS MSCs, SPRY4 was remarkably upregulated following lenti-SPRY4 infection, confirmed by qRT-PCR and western blot assay (Fig. 3a, b). As determined by qRT-PCR, SPRY4 overexpression enhanced mRNA levels of osteogenic marker genes, including *RUNX2*, *ALP*, *IBSP*, *COL1A1* and *OPN* compared to empty vector-infected controls during osteogenic differentiation (Fig. 3c, data from AIS Patient 2 and 3 are not shown). Meanwhile, expression of osteoblast-specific genes at the protein level were also elevated (Fig. 3d). In addition, ALP staining (Fig. 3e), ALP activity (Fig. 3f) and Alizarin red staining (Fig. 3g, h) consistently showed that osteogenic differentiation of AIS MSCs was significantly enhanced in response to SPRY4 overexpression. Therefore, these data revealed that replenishing SPRY4 improves osteogenic differentiation of AIS MSCs in vitro.

### SPRY4 promotes bone formation of MSCs in vivo

**Fig. 4:**
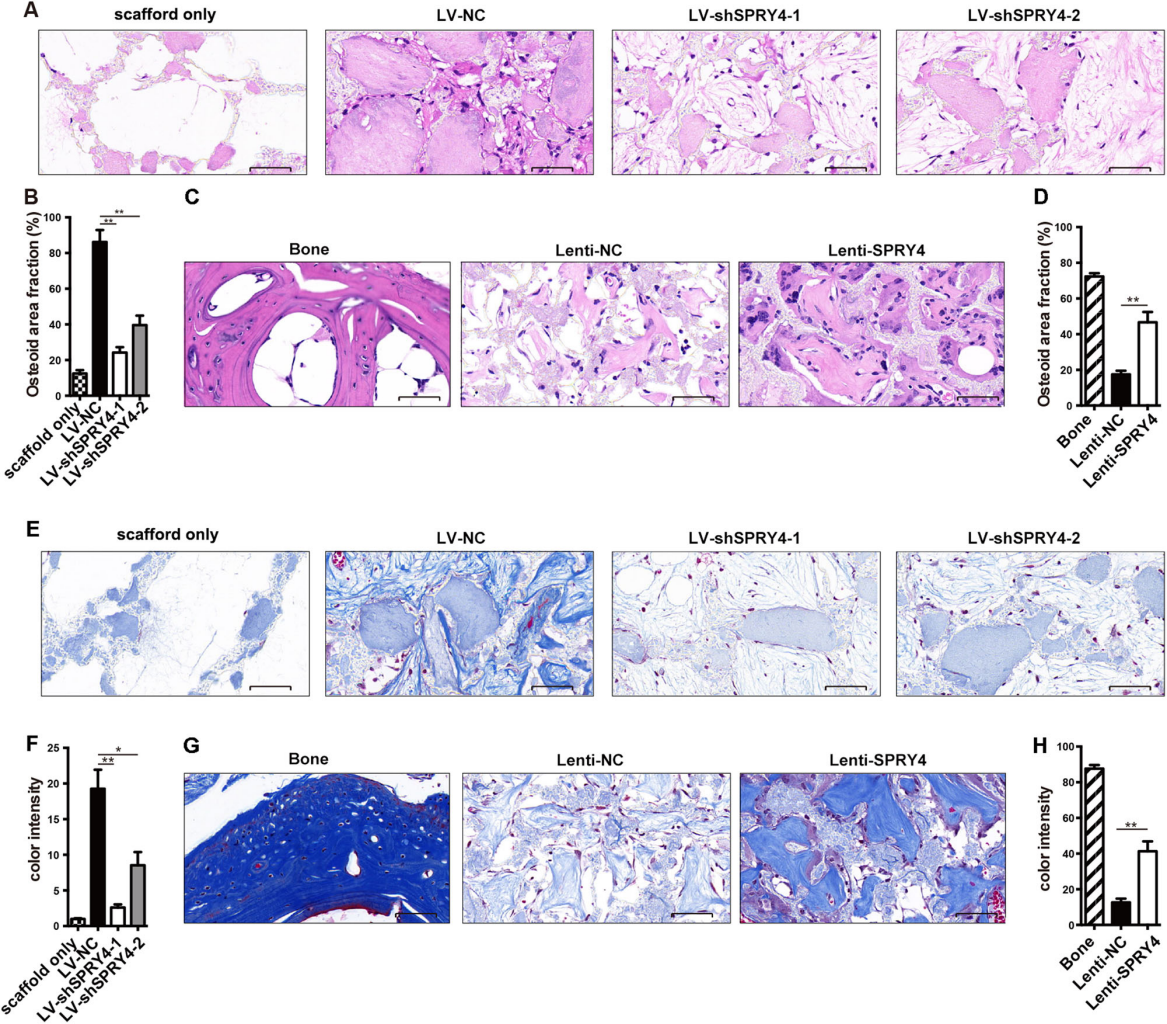
SPRY4 causes heterotopic bone formation of BM-MSCs in vivo. BM-MSCs were infected with lentivirus to silence or overexpress SPRY4. After pre-induction for 3 days, cells were loaded on scaffold materials and implanted into NOD/SCID mice for 10 weeks (*n* = 6 for each group). **a**, **b** Osteoids formed in SPRY4-silenced xenografts (LV-shSPRY4-1 and LV-shSPRY4-2) and control xenografts (LV-NC) were detected by HE staining (**a**), and osteoid area fractions were quantified by Image J (**b**). **c**, **d** Osteoids formed in SPRY4-overexpressed xenografts (lenti-SPRY4) and vector control xenografts (lenti-NC) were detected by HE staining (**c**), and osteoid area fractions were quantified by Image J (**d**). **e**, **f** Collagen deposition in SPRY4-silenced xenografts (LV-shSPRY4-1 and LV-shSPRY4-2) and control xenografts (LV-NC) were detected by Masson staining (**e**), and colour intensity was quantified by Image J (**f**). **g**, **h** Collagen deposition in SPRY4-overexpressed xenografts (lenti-SPRY4) and vector control xenografts (lenti-NC) were detected by Masson staining (**g**), and colour intensity was quantified by Image J (**h**). Scaffolds without cell loading were implanted as negative controls, and femurs from mice were used as positive controls for histological analysis

To further investigate the role of SPRY4 in vivo, we used a model of ectopic bone formation in NOD/SCID mice. BM-MSCs were first incubated in osteogenic medium for 3 days. After pre-induction, MSCs were loaded onto artificial biomimetic mineralized scaffolds overnight and implanted subcutaneously into NOD/SCID mice. Ten weeks later, the implants were harvested for histological analyses. HE staining revealed there were scarce cells in the scaffold only explants, while osteoid formation was obvious in all MSCs plus scaffold groups. SPRY4 depletion clearly decreased osteoid formation in both quantity and size compared to the control groups (Fig. 4a, b). Conversely, SPRY4 overexpression xenografts formed more osteoids than their corresponding controls (Fig. 4c, d). Collagen I is an important component of bone formation, which can be visualized as blue by Masson staining. SPRY4 silencing inhibited collagen generation (Fig. 4e, f), while ectopic expression of SPRY4 enhanced collagen generation (Fig. 4g h). Altogether, these data demonstrate that SPRY4 is positively associated with the bone formation ability of MSCs in vivo.

### SPRY4 functions through activation of the MEK-ERK1/2 pathway

**Fig. 5:**
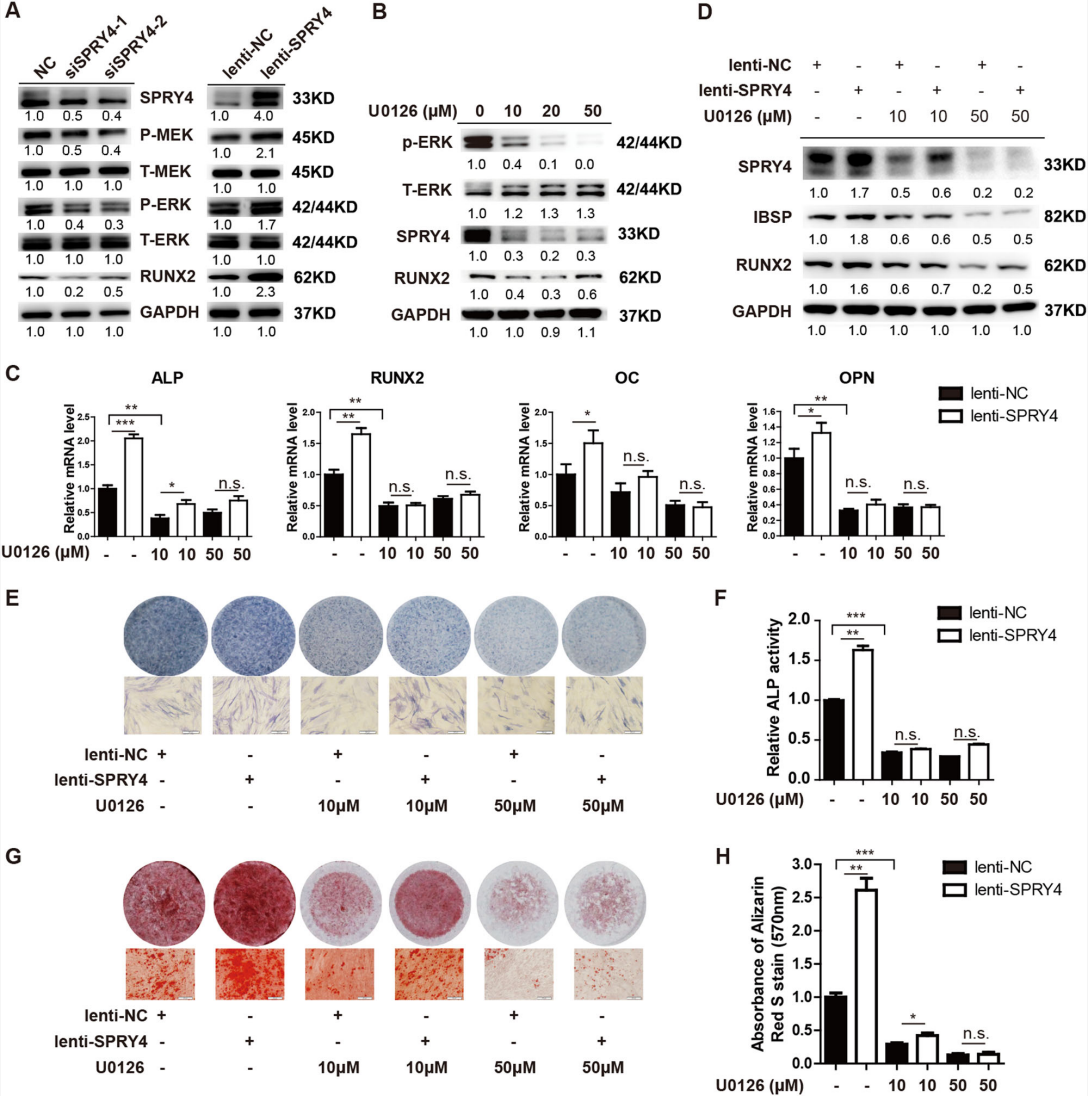
SPRY4 functions via activating MEK- ERK1/2 pathways. **a** Cell extracts were collected after knockdown or overexpression of SPRY4 in BM-MSCs without osteogenic induction. Then, western blot analysis was used to detect P-MEK, P-ERK, T-MEK, T-ERK, SPRY4 and RUNX2. **b** BM-MSCs were treated with ERK1/2 inhibitor U0126 at concentrations of 0, 10, 20 or 50 μM for 24 h to verify its efficiency. Western blot was performed to detect P-ERK, T-ERK, SPRY4 and RUNX2. **c**, **d** qRT-PCR (**c**) and western blot analysis (**d**) detected indicated osteogenic transcription factors and marker genes on day 6 of osteogenic differentiation after overexpressing SPRY4 and treatment with 10 or 50 μM U0126. Data were from three independent experiments using BM-MSCs derived from three healthy donors. **e**, **f** ALP staining and relative ALP activity assays were performed on day 6 of osteogenic differentiation after SPRY4 overexpression and treatment with 10 or 50 μM U0126. Data were from three independent experiments using BM-MSCs derived from 3 healthy donors. **g**, **h** Calcium deposition by Alizarin red S staining and quantification was performed on day 12 of osteogenic differentiation after SPRY4 overexpression and treatment with 10 or 50 μM U0126. Data were from three independent experiments using BM-MSCs derived from three healthy donors. GAPDH was used as a loading control in both qRT-PCR and western blot analyses. Data are shown as the means ± SD

SPRY4, a member of the Sprouty family, is reportedly a regulator of the MAPK pathway. To gain insight into the molecular mechanism by which SPRY4 regulates osteogenic differentiation, we examined activation of MEK-ERK1/2 by western blot. As shown in Fig. 5a, knockdown of *SPRY4* decreased phosphorylation levels of both MEK1/2 and downstream ERK1/2. Conversely, overexpression of SPRY4 increased phosphorylation levels of MEK1/2 and ERK1/2. Level of RUNX2, a previously reported target of the MAPK pathway, was also associated with expression of SPRY4 (Fig. 5a).

Next, we sought to determine whether the MAPK pathway is responsible for the osteogenic promoting effects of SPRY4. MSCs were treated with increasing concentrations of the ERK1/2 inhibitor U0126. As shown in Fig. 5b, 10 μM U0126 was sufficient to block phosphorylation of ERK1/2, resulting in downregulated expression of RUNX2. In line with previous reports, inhibition of ERK1/2 strongly impeded osteogenic differentiation of MSCs (Supplementary Fig. 2). When induced towards osteogenic differentiation, SPRY4 overexpression in MSCs enhanced expression of osteogenic genes (*ALP*, *RUNX2*, *OC* and *OPN)* at the mRNA level (Fig. 5c) and increased expression of osteogenic genes (RUNX2 and IBSP) at the protein level (Fig. 5d). Treatment with U0126 abolished the stimulatory effects of SPRY4 overexpression on osteogenic differentiation (Fig. 5c, d). ALP staining (Fig. 5e), ALP activity (Fig. 5f) and Alizarin red staining (Fig. 5g, h) also confirmed that ERK1/2 inhibition abrogated the osteogenic promoting abilities of SPRY4, as SPRY4 overexpressing MSCs showed equivalent differentiation compared with control MSCs in osteogenic medium supplemented with U0126. Collectively, these data demonstrate that SPRY4-mediated osteogenic differentiation is MEK-ERK1/2-dependent.

### SPRY4 is responsible for melatonin-mediated osteogenic differentiation of MSCs

**Fig. 6:**
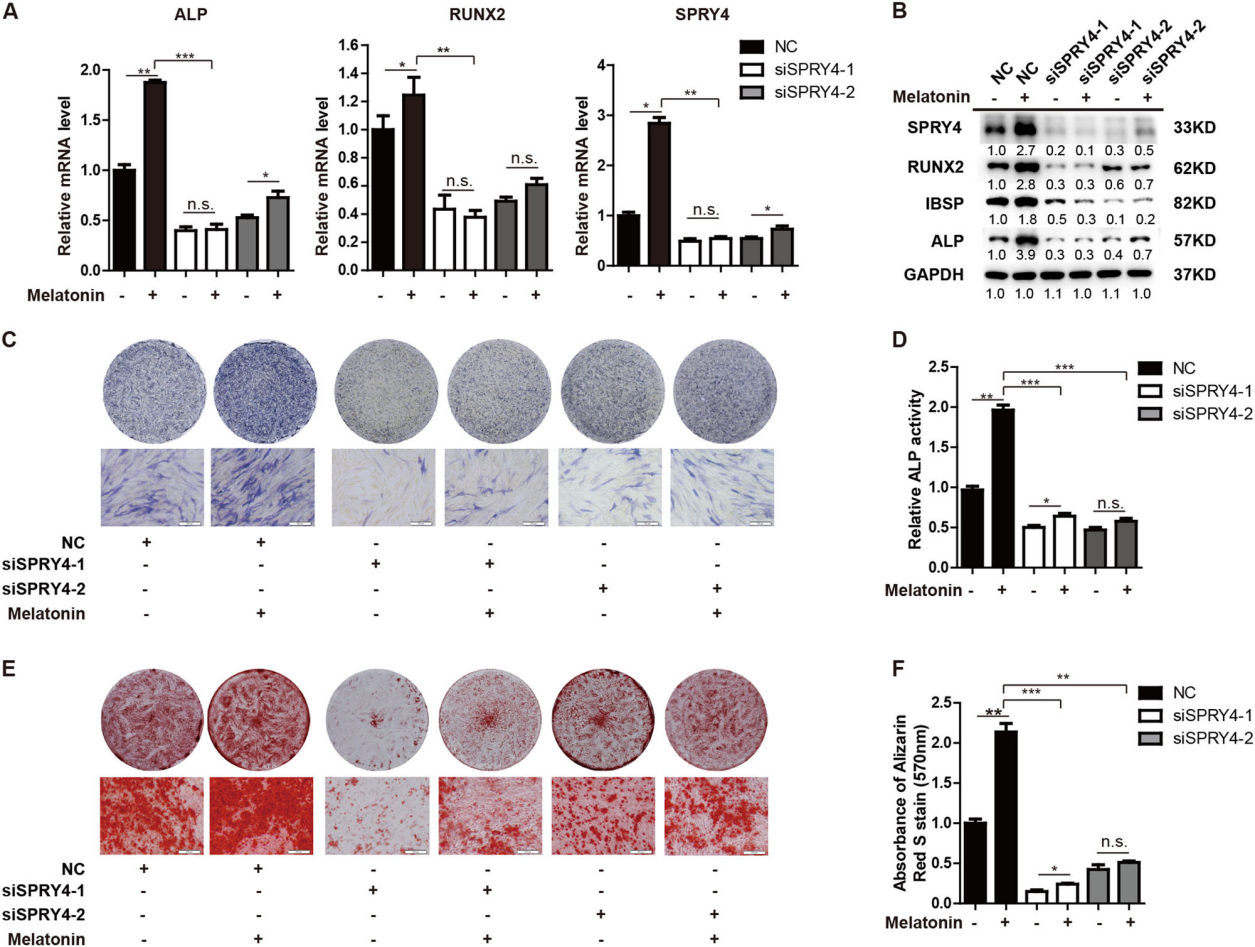
SPRY4 is responsible for melatonin-stimulated osteogenic differentiation of MSCs. **a** SPRY4 was silenced in BM-MSCs, and cells were treated or untreated with 100 μM melatonin during osteogenic induction. qRT-PCR analysis detected *SPRY4, ALP* and *RUNX2* on day 6 of osteogenic differentiation. Data were from three independent experiments using BM-MSCs derived from three healthy donors. **b** Western blot was used to detect SPRY4, OPN and RUNX2 on day 6 of osteogenic differentiation. Data were from three independent experiments using BM-MSCs derived from three healthy donors. **c**, **d** ALP staining and relative ALP activity assays were performed on day 6 of osteogenic differentiation. Data were from three independent experiments using BM-MSCs derived from three healthy donors. **e**, **f** Calcium deposition by Alizarin red S staining and quantification was performed on day 12 of osteogenic differentiation. Data were from three independent experiments using BM-MSCs derived from three healthy donors. GAPDH was used as a loading control in both qRT-PCR and western blot analysis. Data are shown as the means ± SD

As reported in previous studies, melatonin exposure promotes osteoblast differentiation of MSCs, which was validated by increased ALP staining, matrix mineralization, and enhanced expression of osteogenic factors (Supplementary Fig. 3). To examine the role of SPRY4 in melatonin-mediated osteogenic differentiation, we silenced SPRY4 and compared the osteogenic differentiation ability of MSCs induction using osteogenic medium supplemented with vehicle or melatonin. In contrast to controls (NC), SPRY4 silencing in MSCs eliminated the response to melatonin treatment (Fig. 6a, b). Expression of osteogenic factors in SPRY4 depleted MSCs showed no or marginal differences between melatonin treatment and vehicle groups (Fig. 6a, b). ALP staining (Fig. 6c), ALP activity assay (Fig. 6d) and Alizarin red staining (Fig. 6e, f) also demonstrated that knockdown of SPRY4 substantially repressed osteogenic differentiation compared to control MSCs. Moreover, melatonin treatment did not result in improved ALP activity and matrix mineralization in SPRY4 depleted MSCs compared to the vehicle group. These results reveal that SPRY4 is responsible for promoting melatonin-mediated osteoblast differentiation.

### SPRY4 regulates melatonin responses via the MEK-ERK1/2 pathway

**Fig. 7:**
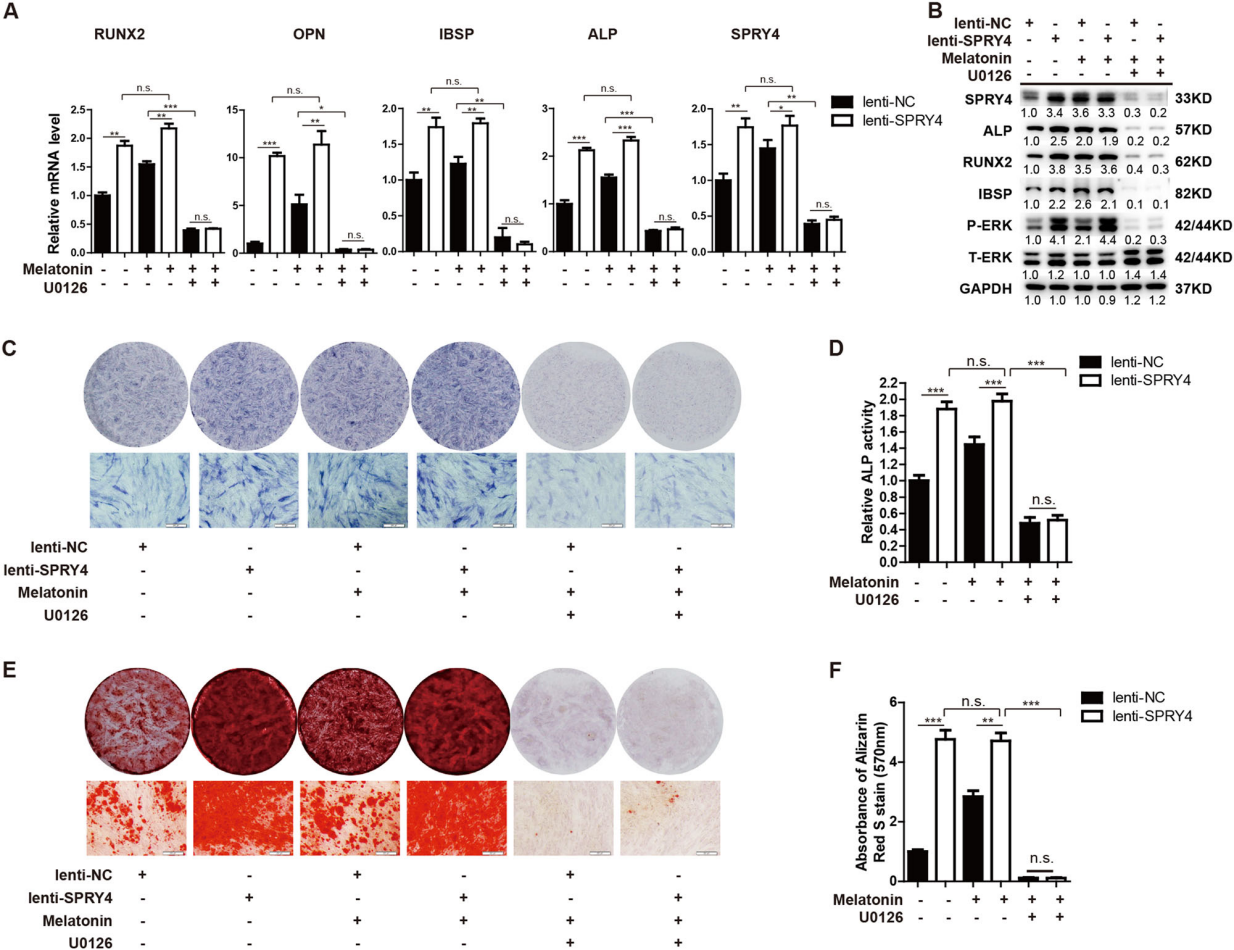
SPRY4 regulates melatonin-mediated responses by MEK-ERK1/2. **a** SPRY4 was overexpressed in BM-MSCs, and cells were treated or untreated with 100 μM melatonin and 10 μM U0126 during osteogenic induction. qRT-PCR analysis detected *SPRY4*, *ALP*, *OPN*, *RUNX2* and *IBSP* on day 6 of osteogenic differentiation. Data were from three independent experiments using BM-MSCs derived from 3 healthy donors. **b** Western blot was used to detect P-ERK, T- ERK, SPRY4, ALP, RUNX2 and IBSP on day 6 of osteogenic differentiation. **c**, **d** ALP staining and relative ALP activity assays were performed on day 6 of osteogenic differentiation. Data were from three independent experiments using BM-MSCs derived from three healthy donors. **e**, **f** Calcium deposition by Alizarin red S staining was performed on day 12 of osteogenic differentiation. Data were from three independent experiments using BM-MSCs derived from three healthy donors. GAPDH was used as loading controls in qRT-PCR and western blot analysis. Data are shown as the means ± SD

To confirm whether SPRY4 is involved in the melatonin signalling pathway through the MEK-ERK1/2 cascade, we assessed the effects of melatonin exposure and SPRY4 overexpression on osteogenesis using the ERK1/2 inhibitor U0126. As shown in Fig. 7a, SPRY4 overexpression enhanced the expression of osteogenic genes (*RUNX2*, *OPN*, *IBSP* and *ALP*) compared to NC MSCs. In SPRY4 overexpressing MSCs, melatonin treatment did not further increase expression of osteogenic genes compared to vehicle treatment (Fig. 7a, b). These data suggest that melatonin and SPRY4 might exert effects on the same pathway. Inhibition of the MEK-ERK1/2 pathway abrogated both melatonin treatment- and SPRY4 overexpression-induced upregulation of osteogenic differentiation. ALP staining (Fig. 7c), ALP activity assay (Fig. 7d) and Alizarin red staining (Fig. 7e, f) also demonstrated that melatonin treatment could not further enhance ALP activity or matrix mineralization in SPRY4 overexpressing MSCs, and the ERK inhibitor U0126 repressed osteogenic differentiation caused by both melatonin treatment and SPRY4 overexpression. Collectively, these results suggest that SPRY4-mediated melatonin-stimulated osteogenic differentiation occurs via the MEK-ERK1/2 signalling pathway.

## Discussion

Low bone mass and abnormal melatonin responses are reportedly associated with AIS pathology. As a progenitor of osteoblast and chondroblasts, AIS MSCs have been shown to exhibit decreased capacity for osteogenic differentiation and aberrant melatonin signalling. In this study, we showed that SPRY4 is significantly downregulated in AIS bone marrow derived MSCs compared to cells from healthy donors. SPRY4 knockdown suppressed osteogenic differentiation of normal MSCs, while overexpression of SPRY4 improved osteogenic differentiation of AIS MSCs. Moreover, we found that SPRY4 positively modulates melatonin-induced activation of MEK/ERK1/2 and the subsequent downstream target RUNX2, leading to osteogenic differentiation of MSCs.

Sproutys (SPRYs) are highly conserved protein families well known as negative regulators of FGF signalling that fine-tune RTK signalling. RTK signalling is critical for embryonic development, as well as disease pathogenesis, such as congenital syndromes and cancer^36,37^. Interruption of FGF signalling by gene-environment interaction has been proven to be associated with the aetiology of congenital scoliosis^38^. To date, four Sprouty members (SPRY1–4) have been identified in mammals. Knockout mice have been generated to characterize the function of SPRYs in a few studies and are reviewed by others^36,39^. SPRY2 and SPRY4 double knockout mice are embryonic lethal by E12.5 with craniofacial and limb morphogenesis abnormalities, supporting that loss of SPRYs leads to hyperactivation of FGF signalling^40^.

In contrast to the inhibitory effect of SPRY4 on FGF/MAPK signalling, herein, we report an active effect of SPRY4 on melatonin/MAPK signalling. Knockdown of SPRY4 decreased phosphorylation of MEK/ERK1/2, while overexpression of SPRY4 enhanced phosphorylation of MEK/ERK1/2. Indeed, previous studies reported that SPRYs not only negatively but also positively, regulate ERK activation depending on the type of growth factor stimulation and cell type involved^41^. SPRY2 has been shown to suppress FGF, PDGF and VEGF induction, while not inhibiting EGF-induced activation of ERK^42^. Consistently, Joo et al reported that SPRY2 knockout mice exhibit defective chondrogenesis and endochondral bone formation with decreased RTK signalling^43^. We treated BM-MSCs with FGF, EGF and melatonin separately. We found that SPRY4 knockdown enhanced MAPK signal in response to FGF treatment, while SPRY4 knockdown inhibited MAPK signal in response to EGF and melatonin treatment (Supplementary Fig. 4). Our findings suggested that SPRY4 regulates the MAPK pathway dependent on the type of ligands in BM-MSCs. Mechanistically, SPRYs can bind various interaction partners, such as c-Cbl, Grb2 or Raf-1, differentially regulating the RTK signalling pathway^44^. The effects of melatonin are dependent on four possible pathways, including G-protein-coupled membrane receptors (MT1/MT2), orphan nuclear receptor (ROR/RZR), intracellular proteins such as calmoduline, and antioxidative effects^45,46^. Detailed molecular mechanisms underlying the active functions of SPRY4 in response to melatonin remain to be further studied.

In this study, melatonin treatment resulted in activation of the MEK/ERK1/2 pathway and upregulation of SPRY4 at mRNA and protein levels, suggesting that SPRY4 is a target of the MEK/ERK1/2 cascade at the transcriptional level. Of note, blocking the MEK-ERK1/2 pathway with U0126 decreased expression of SPRY4, even in MSCs with forced ectopic expression of SPRY4. These data suggest that, in addition to transcriptional regulation, other mechanisms, such as protein translation, post-translation modification, or stability, must exist by which ERK1/2 may regulate the expression of SPRY4.

In this study, we demonstrated that RUNX2 is a downstream target of SPRY4 in normal BM-MSCs via the MEK/ERK1/2 pathway. ERK inhibition using U0126 remarkably repressed osteoblast differentiation induced by both SPRY4 overexpression and melatonin exposure. RUNX-2 is a master regulator of osteoblast differentiation and bone formation, and it can directly stimulate transcription of many other osteogenic genes, including osteocalcin, type I collagen and osterix^47,48^. Several pathways leading to RUNX2 activation, such as BMP, bFGF and IGF, were found to converge at the MAPK cascade^32,49,50^. Moreover, several recent studies demonstrated that there are positive correlations between low RUNX2 expression and BMD in AIS patients^51,52^. These studies are consistent with our results, suggesting that MAPK signalling plays a key role in bone formation and may be implicated in AIS pathogenesis.

The role of melatonin deficiency in the aetiology or pathology of AIS has long been controversial. Supporting evidence comes from observations that pinealectomized chickens, bipedal rats and C57BL/6J mice develop idiopathic-like scoliosis. In addition, AIS mostly occurs during puberty when melatonin excretion remains relatively high during the lifetime. Opposing views are mainly based on conflicting observations regarding human melatonin levels between AIS patients and healthy controls. Recently, some researchers have postulated that instead of deficiency in melatonin levels, a defect in melatonin signalling activity may contribute to AIS etiopathology. The melatonin receptor 1B gene (MTNR1B) has associated with the occurrence of AIS in both gene polymorphism^53^ and abnormal protein expression^54^. Protein kinase C delta, which regulates phosphate of G inhibitory proteins normally associated with the melatonin receptor, was found to be dysfunctional in osteoblasts from AIS patients^16,17^. Consistent with these reports, we found that SPRY4, a member responsible for melatonin-stimulated MEK/ERK1/2 activation, is dysregulated in AIS. Intercellular protein dysfunction in transduction machinery could result in similar effects as melatonin deficiency. Loss of SPRY4 in MSCs markedly repressed osteogenic differentiation, and melatonin further augmented the differences between SPRY4 knockdown and control groups. Our study may help elucidate the contradictions concerning the role of melatonin in AIS etiopathology and highlight the complexity and heterogeneity associated with AIS. However, based on current data, we cannot conclude whether low expression of SPRY4 is a cause or consequence of aberrant melatonin/MAPK signalling pathway in AIS MSCs. Further investigation is needed to answer these questions.

Previous studies have suggested that AIS is associated with uncoupled endochondral-membranous bone formation during the growth spurt^6,55,56^ Longitudinal growth, achieved mainly by endochondral ossification, is more rapid than circumferential growth in vertebral bodies. In contrast, circumferential growth, which is achieved by membranous ossification, is relatively slower in AIS patients. Dissociation between longitudinal and circumferential growth, resulting in anterior column overgrowth in the scoliotic spine, may contribute to the development of AIS^6^. In addition, since both endochondral and intramembranous ossification begin with MSC condensation and developmental programmes, MSC abnormalities surely impair the balance between these two pathways. Our findings suggest that SPRY4-mediated aberrant melatonin/MAPK signalling in AIS MSCs may be associated with the imbalance of membranous and endochondral ossification of MSCs during the growth period in AIS patients. Certainly, further studies are still required to elucidate these mechanisms.

In summary, we provide evidence that SPRY4, an RTK regulator, plays a critical role in the regulation of osteogenic differentiation and melatonin response. Deregulation of SPRY4 occurring in AIS MSCs might contribute to the low bone mineral density and abnormal skeletal growth observed in AIS. Our findings provide new insights for understanding the effects of melatonin on AIS aetiology and highlight the importance of MSCs in AIS pathogenesis.

## Supplementary information

Supplementary figures and table
