# Supplementary material for: SPRY4 is responsible for pathogenesis of adolescent idiopathic scoliosis by contributing to osteogenic differentiation and melatonin response of bone marrow-derived mesenchymal stem cells
Source: Cell Death Dis. 2019 Oct 23;10(11):805. doi: 10.1038/s41419-019-1949-7 (PMC6811559; doi:10.1038/s41419-019-1949-7)
Supplement: Supplementary file 1 — Supplementary figures and table [file 41419_2019_1949_MOESM1_ESM.docx]

**Supplementary Figures**


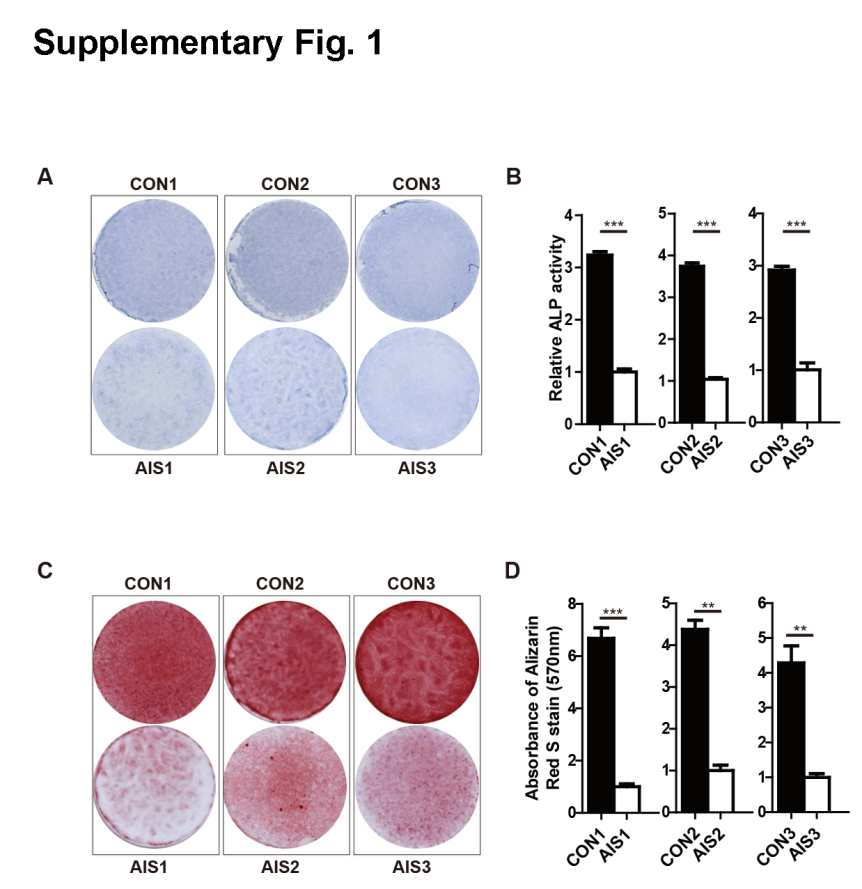


**Supplementary Figure 1. Osteogenic differentiation potential is decreased in AIS patients**

(A, B) AIS BM-MSCs from three patients and CON BM-MSCs from three non-AIS patients were induced towards osteogenic differentiation. ALP staining and relative ALP activity assays were performed on day 6 of osteogenic differentiation. (C, D) Calcium deposition by Alizarin red S staining and quantification was performed on day 12 of osteogenic differentiation. Data are shown as the means ± SD.


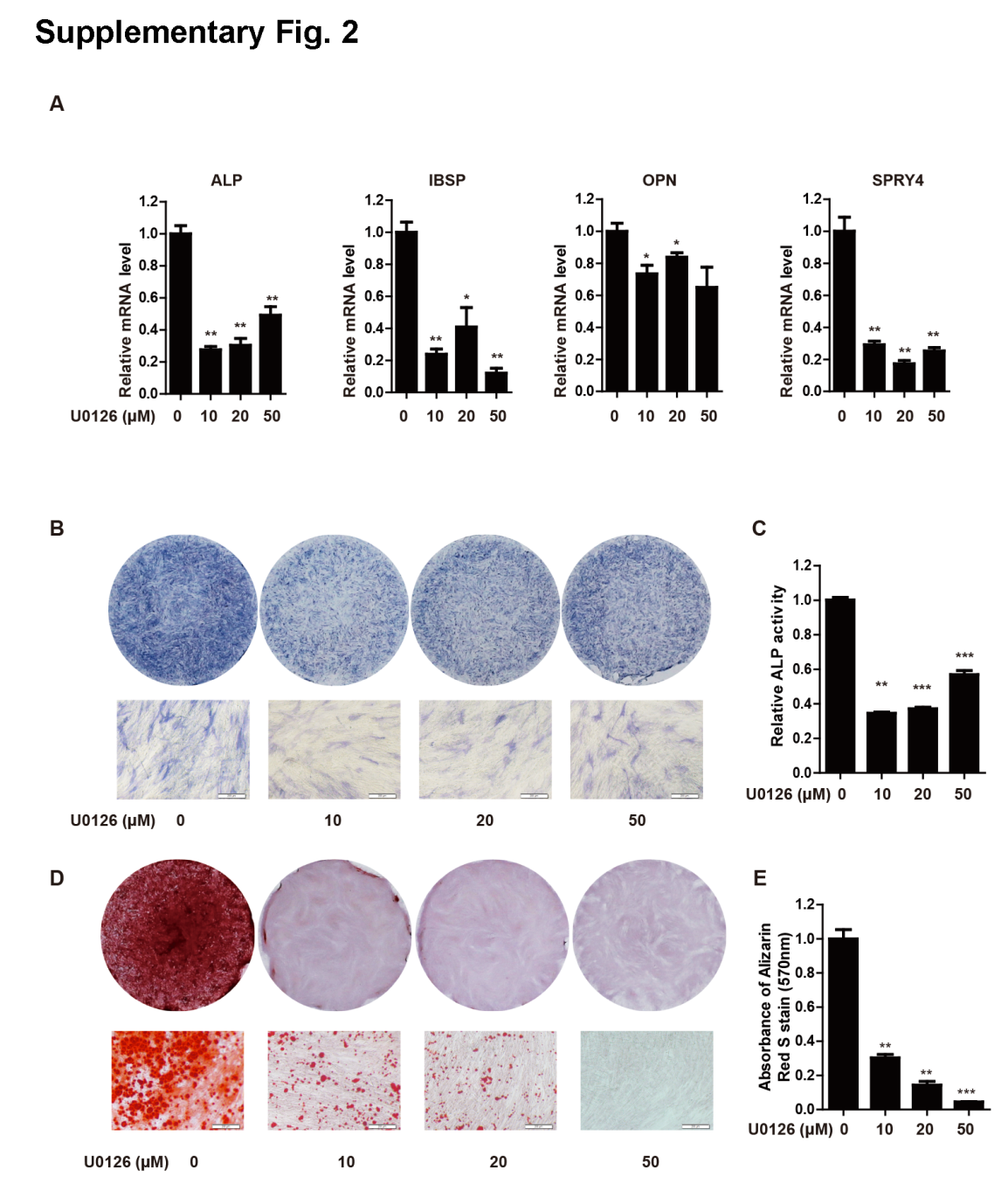


**Supplementary Figure 2. MEK1-ERK1/2 pathway inhibition suppresses osteogenic differentiation.** (A) ERK inhibitor U0126 was used to block the MEK-ERK pathway with different concentrations (0, 10, 20, 50 μM) added to osteogenesis induction medium. qRT-PCR analysis detected osteogenic transcription factors and marker genes on day 6 of osteogenic differentiation. (B, C) ALP staining and relative ALP activity assays were performed on day 6 of osteogenic differentiation. (D, E) Calcium deposition by Alizarin red S staining and quantification was performed on day 12 of osteogenic differentiation. GAPDH was used as a loading control in both qRT-PCR and western blot analyses. Data are shown as the means ± SD.


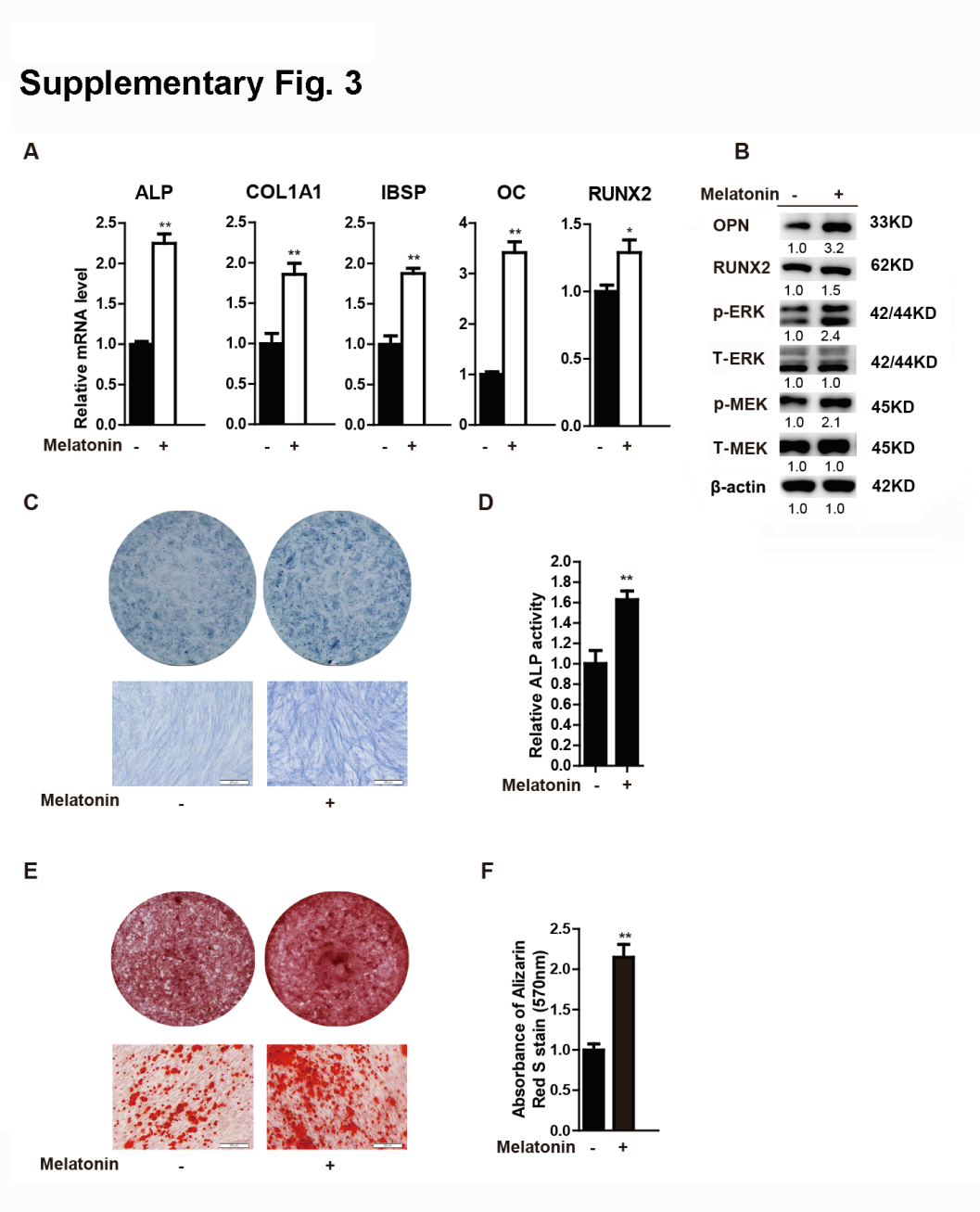


**Supplementary Figure 3. Melatonin promotes osteogenic differentiation of BM-MSCs**

(A, B) BM-MSCs were induced to osteogenic differentiation, 100 µM melatonin was added to osteogenesis induction medium. qRT-PCR (A) and western blot (B) analysis detected osteogenic transcription factors and marker genes on day 6 of osteogenic differentiation. (C, D) ALP staining and relative ALP activity assays were performed on day 6 of osteogenic differentiation. (E, F) Calcium deposition by Alizarin red S staining and quantification was performed on day 12 of osteogenic differentiation. GAPDH or β-actin was used as a loading control in qRT-PCR and western blot analyses. Data are shown as the means ± SD.


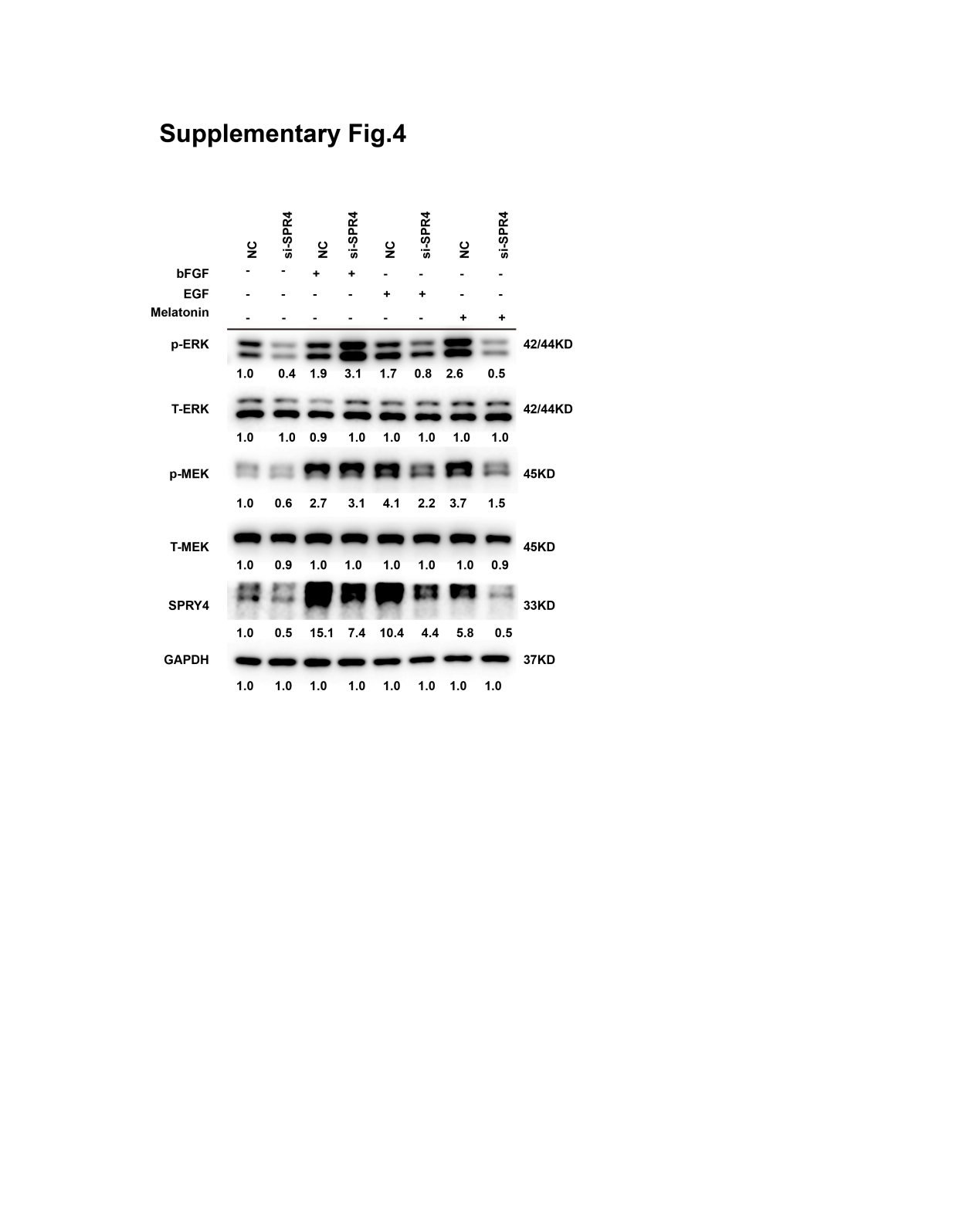


**Supplementary Figure 4. SPRY4 regulates MEK1-ERK1/2 pathway in a ligand-dependent way.** SPRY4 was silenced in BM-MSCs from healthy donors by siRNA. After 24 h, BM-MSCs were treated with bFGF (100 ng/ml), EGF (100 ng/ml), or melatonin (100 µM) for another 24 h, P-ERK, T-ERK, P-MEK, T-MEK, SPRY4 and GAPDH were detected by Western blot.

**Supplementary Table 1. All primers used in this study**

| **Gene** | **Primer sequence** | **Product size (bp)** |
| --- | --- | --- |
| SPRY4 | F: 5’-CTGACCAACGGCTCTTAGAC-3’ | 196 |
|  | R: 5’-GATGCACACTCCTTGCATTTAC-3’ |  |
| RUNX2 | F: 5’-TGTCATGGCGGGTAACGAT-3’ | 147 |
|  | R: 5’-AAGACGGTTATGGTCAAGGTGAA-3’ |  |
| ALP | F: 5’-CCACGTCTTCACATTTGGTG-3’ | 196 |
|  | R: 5’-AGACTGCGCCTGGTAGTTGT-3’ |  |
| OPN | F: 5’-ACTCGAACGACTCTGATGATGT-3’ | 224 |
|  | R: 5’-GTCAGGTCTGCGAAACTTCTTA-3’ |  |
| OC | F: 5’-GGCAGCGAGGTAGTGAAGA-3’ | 148 |
|  | R: 5’-CCTGAAAGCCGATGTGGT-3’ |  |
| COL1A1 | F: 5’-CCCAAGGAAAAGAAGCACGTC-3’ | 109 |
|  | R: 5’-AGGTCAGCTGGATAGCGACATC-3’ |  |
| IBSP | F: 5’-CCCCACCTTTTGGGAAAACCA-3’ | 109 |
|  | R: 5’-TCCCCGTTCTCACTTTCATAGAT-3’ |  |
| GAPDH | F: 5’-GGTCACCAGGGCTGCTTTTA-3’ | 195 |
|  | R: 5’-GGATCTCGCTCCTGGAAGATG-3’ |  |
| NC | r(UUCUCCGAACGUGUCACGU)dTdT |  |
| siSPRY4-1 | r(GACCAGCCAUGUGGAGAAU)dTdT |  |
| siSPRY4-2 | r(UCAACUAUGGCACGUGCAU)dTdT |  |
